# P-1296. Community-acquired methicillin-resistant *Staphylococcus aureus* infections: 1 year of surveillance in 6 pediatric centers

**DOI:** 10.1093/ofid/ofae631.1477

**Published:** 2025-01-29

**Authors:** Angela Gentile, Julia Bakir, Maria Veronica Firpo, Ximena S Juarez, Aldo Cancellara, Gabriela Tapponnier, Agostina Tinnirello, Oscar Lopez, Leandro Lopez, Maria Natalia Pejito, Santiago Lopez Papucci, Gabriela Ensinck

**Affiliations:** Ricardo Gutierrez Children's Hospital, Buenos Aires, Ciudad Autonoma de Buenos Aires, Argentina; Ricardo Gutierrez Children's Hospital, Buenos Aires, Ciudad Autonoma de Buenos Aires, Argentina; Hospital del Niño Jesus, Tucuman, Tucuman, Argentina; Hospital de Niños P. de Elizalde, Buenos Aires, Buenos Aires, Argentina; Hospital de Niños P. de Elizalde, Buenos Aires, Buenos Aires, Argentina; Hospital Nacional Alejandro Posadas, Buenos Aires, Buenos Aires, Argentina; Hospital Nacional Alejandro Posadas, Buenos Aires, Buenos Aires, Argentina; Hospital Pediatrico Fernando Barreyro, Misiones, Misiones, Argentina; Hospital Pediatrico Fernando Barreyro, Misiones, Misiones, Argentina; Hospital de Niños Ricardo Gutierrez, CABA, Ciudad Autonoma de Buenos Aires, Argentina; Hospital de Niños Victor Vilela, Rosario, Santa Fe, Argentina; Hospital de Niños Victor Vilela, Rosario, Santa Fe, Argentina

## Abstract

**Background:**

Community-acquired methicillin-resistant *Staphylococcus aureus* (CA-MRSA) infections is emerging in the world and can have a serious evolution. The objective of this study were to study the hospitalization rate, the clinical-epidemiological and bacteriological pattern and the case fatality risk factors of CA-MRSA infection.

**Methods:**

Cross-sectional observational epidemiological study, with prospective data capture. All patients ≤ 15 years of age with community-acquired *Staphylococcus aureus* (CA-SA) infection hospitalized in 6 pediatric sentinel units, between January and December 2023, were included. The rate of CA-MRSA cases per 10,000 hospital discharges was calculated. with 95 % confidence interval (95 % CI).

**Results:**

Of a total of 201 patients with CA-SA infection, 131 (65.2 %) were CA-MRSA. The global hospitalization rate for CA-MRSA cases was 27.8 (23.3-32.9), with a higher rate in < 2 years 37,7 (27,9-49,8). Of the 131 cases: 56.5 % were male, median age 48.8 months (IQR 16.4-90.4), ≥ 2 years 64.9%, complete vaccination card 77.0 %, low socioeconomic level 54.2 %, overcrowding 15.3 %, underlying disease 29.0 %, passive smoking 24.4 %. The clinical presentations were (n; %): skin and soft tissue infection (SSTI 101; 77.1 %), arthritis (17; 13.0 %), osteomyelitis (12; 9.2 %), pneumonia (16; 12.2 %; 14 with pleural effusion and 3 necrotizing), sepsis (11; 8.4 %), bacteremia (4; 3.1 %), meningitis (1; 0.8 %). Conventional microbiological diagnosis was performed in 130 cases and molecular diagnosis in one. 4 cases had coinfection with group A beta hemolytic *Streptococcus*. Antibiotic resistance was for erythromycin 43.1 %, clindamycin 26.0 %, gentamicin 19.9 %, TMP-SMX 1.5 %. All strains were susceptible to vancomycin. Fatality rate: 1.5 % (2/131). No deceased case had an underlying disease. 18.2 % (2/11) of sepsis cases died (2-tailed Fisher exact p 0.006), however, there was no significant association in the multivariate analysis.

**Conclusion:**

The high burden of CA-MRSA infection, the most common clinical form of SSTI, and the high profile of antibiotic resistance, highlight the importance of continuous clinical surveillance and alert for these infections.

**Disclosures:**

**All Authors**: No reported disclosures

